# What do Health/Mental Health Professionals Have to do With Racial Discrimination?

**DOI:** 10.1192/j.eurpsy.2022.64

**Published:** 2022-09-01

**Authors:** L. Küey

**Affiliations:** Istanbul Bilgi University, Department For Psychology, Istanbul, Turkey

## Abstract

There is a growing evidence that social determinants of health influence the health outcomes. These non-medical factors, i.e., social determinants of health / mental health, are defined as the conditions in which people are born, grow, live, work and age, including the factors shaping these conditions. They either have direct effects on health and ill health or work as mediators. In this respect, racial discrimination is a fundamental social determinant of ill health / mental health and health inequalities. A strong correlation between reported experiences of racial discrimination and poor general health and poor mental health has been reported. Besides, racial discrimination may lead to risk taking behaviors increasing poor health / mental health especially in vulnerable disadvantaged populations. A leading factor mediating the negative effects of any biopsychosocial factor on mental ill health is the degree of discrimination. Furthermore, racial discrimination is one of the processes explaining and reinforcing racial disparities in health and ill health. From a conceptual point of view, racial discrimination and its effects on ill health could be discussed in the context of the issue of othering and related dehumanization and violence. Psychiatrists and mental health workers have accumulated considerable knowledge and experience on understanding and overcoming some of the consequences of racial discrimination, especially via anti-stigma studies. The unfair and avoidable influences of racial discrimination on mental health are neither fated nor inevitable. As Gramsci had said, we have the pessimism of the intellect and optimism of the will.

**Disclosure:**

No significant relationships.

